# Cytokine and Growth Factor Activation In Vivo and In Vitro after Spinal Cord Injury

**DOI:** 10.1155/2016/9476020

**Published:** 2016-06-23

**Authors:** Elisa Garcia, Jorge Aguilar-Cevallos, Raul Silva-Garcia, Antonio Ibarra

**Affiliations:** ^1^Facultad de Ciencias de la Salud, Universidad Anáhuac, 52786 Huixquilucan, MEX, Mexico; ^2^Centro de Investigación en Ciencias de la Salud (CICSA), Facultad de Ciencias de la Salud, Universidad Anáhuac, 52786 Huixquilucan, MEX, Mexico; ^3^Unidad de Investigación Médica en Inmunología, Hospital de Pediatría, Centro Médico Nacional Siglo XXI, IMSS, 52786 México, MEX, Mexico

## Abstract

Spinal cord injury results in a life-disrupting series of deleterious interconnected mechanisms encompassed by the primary and secondary injury. These events are mediated by the upregulation of genes with roles in inflammation, transcription, and signaling proteins. In particular, cytokines and growth factors are signaling proteins that have important roles in the pathophysiology of SCI. The balance between the proinflammatory and anti-inflammatory effects of these molecules plays a critical role in the progression and outcome of the lesion. The excessive inflammatory Th1 and Th17 phenotypes observed after SCI tilt the scale towards a proinflammatory environment, which exacerbates the deleterious mechanisms present after the injury. These mechanisms include the disruption of the spinal cord blood barrier, edema and ion imbalance, in particular intracellular calcium and sodium concentrations, glutamate excitotoxicity, free radicals, and the inflammatory response contributing to the neurodegenerative process which is characterized by demyelination and apoptosis of neuronal tissue.

## 1. Introduction

Traumatic spinal cord injury (SCI) is a complex, life-disrupting medical condition due to the detrimental effects on social, familiar, and personal life, which include in the majority of cases permanent paralysis due to the low regenerative capacity of the central nervous system (CNS).

SCI triggers a series of interconnected mechanisms that can be divided into the primary and secondary injury. The direct and immediate physical disruption of neurons, glial cells, and blood vessels makes up the primary injury. In turn, the secondary injury consists of a cascade of autodestructive cellular and molecular mechanisms that exacerbate the primary injury and lead to an enlargement of the initial area of trauma [1–4]. Several mechanisms take part in this latter phase of the injury, including vascular disruption, increased blood-spinal cord barrier permeability, ionic dysregulation, edema, excessive intracellular calcium concentration, glutamate excitotoxicity, lipid peroxidation, an autoreactive inflammatory reaction, and apoptosis [5]. Ultimately, the sum of these processes causes cell death, demyelination, and axonal degeneration at the epicenter of injury and the surrounding regions.

These cellular and molecular changes that occur early after SCI alter gene expression profiles, which is characterized by a significant upregulation of genes with roles in transcription, inflammation, and signaling proteins [6]. Evidence suggests that the consequent inflammation mediated by cytokines, growth factors, and related molecules plays a role in both the damage and repair of injured neural tissue [7–9]. The critical balance between these processes plays a major participation in the progression and outcome of a neurodegenerative process [10].

Cytokines encompass a large family of small signaling proteins involved in intercellular communication that are normally associated with the immune response and its modulation but have pleiotropic effects in the physiology of health and disease including cellular growth, survival, and differentiation. These molecules, which can be classified as peptides, proteins, or glycoproteins, are secreted by numerous cells and can be grouped into a proinflammatory or anti-inflammatory category on the basis of the final balance of their effects [10]. Subsequently, growth factors are proteins synthesized by a wide variety of cells that stimulate cellular survival, chemotaxis, proliferation, and differentiation [11, 12]. The aim of this review is to expose the role of cytokines and growth factors within the pathogenesis of SCI, since the study of these molecules could bring to light novel potential therapeutic targets that could reduce the degenerative processes that occur after SCI.

## 2. Autodestructive Mechanisms after Spinal Cord Injury

### 2.1. Disruption of the Blood Spinal Cord Barrier

The blood-CNS vascular barriers consist of complexes of adherence junction proteins and tight junctions, astrocyte endfeet, perivascular microglia, pericytes, and continuous capillary endothelial cells embedded in the basement membrane that separate and protect the CNS from metabolites and neurotoxic substances present in the systemic circulation [13–15]. This infrastructure allows the blood brain barrier (BBB) and blood spinal cord barrier (BSCB) to regulate the transport of molecules, the interaction between the CNS and the immune system, and helps maintaining homeostasis in the brain and spinal cord.

One of the earliest events ensuing traumatic SCI is the disruption of the BSCB by a mechanical force that destroys neural tissue and tears neuronal and endothelial cell membranes [5]. The resulting inflammatory response disturbs the microenvironment of the spinal cord, alters vascular permeability, facilitates the entry of peripheral immune cells, and exposes the adjacent noninjured tissue to potentially noxious molecules [16, 17]. These molecules include early inflammatory cytokines such as interleukin 1*β* (IL-1*β*) and tumor necrosis factor *α* (TNF*α*); in addition, they might include nitric oxide (NO^•^), reactive oxygen species (ROS), elastase, and matrix metalloproteinase-9 (MMP-9) [17]. The importance of the BSCB is evidenced by the positive correlation between increased barrier disruption and improved motor locomotion 14 days after SCI [18–20].

An additional consequence of such disruption is a series of regulatory changes in the transport systems for selective cytokines that may induce regenerative or destructive effects. In particular, there is an upregulation of the transport system of TNF*α* after SCI that remains saturable despite BSCB disruption. The increase of TNF*α* takes place before other cytokines in SCI and is mediated by the receptor-based transport composed by TNFR1 (p55) and TNFR2 (p75) [21]. TNF*α* has a role in inflammation, myelin destruction, apoptotic neuronal cell death, and astrocyte toxicity. Nevertheless, this cytokine is also capable of stimulating neurite outgrowth, secretion of growth factors, and tissue remodeling [21]. It has been suggested that TNF*α* has a dual role: deleterious in the acute phase, but beneficial in the chronic phase after SCI [22].

Similarly, leukemia inhibitory factor (LIF) utilizes a transport system mediated by LIFR (gp190), which is upregulated by barrier disruption, but remains saturable despite this event [21, 23]. LIF is involved in the activation of microglia/macrophages and in the proinflammatory response in SCI [24]. Contrastingly, LIF has been shown to prevent oligodendrocyte apoptosis in mice with SCI after overhemisection, notably contralateral to the spinal cord lesion, through the induction of the JAK/STAT and Akt signaling pathways as well as by potentiating the expression of the antiapoptotic molecule, cIAP2. Reduced oligodendrocyte apoptosis after SCI with LIF administration resulted in a substantial decrease in demyelination shown by the preservation of lamellated myelin surrounding viable axons and deposition of the degraded myelin basic protein. The data suggest that LIF signals survival in oligodendrocytes after SCI, prevents the secondary wave of demyelination, and thereby reduces inhibitory myelin deposits and enhance locomotor recovery [25].

### 2.2. Edema and Ion Imbalance

Immediately after contusive SCI, the rupture of the blood-CNS barrier causes water to accumulate in the extracellular compartment and results in the production of neural tissue edema [26, 27]. This is a process that may aggravate the initial injury and result in paraplegia or even death [13]. The subsequent increment in vascular permeability and the formation of edema could also be in part mediated by the vascular endothelial growth factor (VEGF) and proto-oncogene tyrosine-protein kinase (Src/c-Src) which exists downstream of VEGF [28]. It is worth noting that administration of VEGF has resulted in an increase in permeability of the BSCB from the acute to chronic phase, which is interesting since it is regarded to be a component involved in angiogenesis, neurogenesis, and locomotor recovery [29].

As the secondary injury progresses, this fluid accumulation in the CNS becomes characterized by ionic imbalance, which consists of an increase in the intracellular concentration of Na^+^ and Ca^2+^, in conjunction with an elevated extracellular concentration of K^+^ and Mg^+^ [30–32]. Consequently, the Na^+^ and Ca^2+^ ions attract water molecules into the cell and cause edema. The resulting fluid accumulation then propels the compression of adjacent tissues and the development of ischemia, which leads to more autodestructive phenomena such as free-radical production, lipid peroxidation, and inflammation. It is important to note that the edema that occurs after contusive SCI is directly related to the initial trauma and motor dysfunction experienced by the affected individual [27, 33].

Astrocytes are the principal regulators of water transport in the CNS, where they are additionally linked to the maintenance of ion homeostasis, spatial buffering of extracellular potassium, calcium signal transduction, adult neurogenesis, and neurotransmitter uptake and release [34–36]. A molecule expressed in astrocyte endfeet, astrocyte processes, and the basolateral membrane of ependymal cells is Aquaporin 4 (AQP4), the predominant water channel in the CNS [36]. Recent studies indicate that AQP4 regulates the before-mentioned astrocytic functions [36].

Moreover, the absence of AQP4 has been shown to reduce proinflammatory cytokines in astrocytes such as TNF*α* and interleukin-6 (IL-6) after CNS injury [37]. It is important to mention that the role of AQP4 in the resolution of edema is still under debate [37]. Nevertheless, evidence demonstrates that AQP4 has an essential role in the formation and distribution of edema and that it is intrinsically involved in the development of the inflammatory process after an insult to the CNS [37].

On the other hand, neurons regulate synaptic transmission and neural plasticity by the activation of membrane receptors and channels in adjacent neurons. Released neurotransmitters can bind to inhibitory (GABA)ergic receptors or excitatory glutamate receptors such as amino-3-hydroxy-5-methyl-4-isoxazolepropionic acid (AMPA), N-methyl-D-aspartate (NMDA), kainate, and metabotropic receptors [38]. In the locomotor networks of the spinal cord, Ca^2+^ activated, apamin-sensitive K^+^ channels (SK) control the firing of constituent neurons and regulate the locomotor rhythm. Voltage-gated Ca^2+^ channels (VGCCs), such as N-type Ca^2+^ channels, are considered the main activators of SK channels [39], which during early development play a role in neurite outgrowth and functional neuromuscular synapse organization [40]. NMDA receptors, besides controlling evoked neurotransmitter release, also play a role in the activation of SK channels in dendrites [39, 40]. SK channels have been found to regulate hippocampal synaptic plasticity, learning, and memory, particularly SK2 channels [41]. Synaptic transmission involves Ca^2+^ and employs calmodulin (CaM) dependent kinases (CaMKIIV), protein kinase C, protein kinase A, IP3 kinase, Ca^2+^-dependent phosphatase calcineurin B, cyclic AMP phosphodiesterase, adenylyl cyclase, lCa^2+^-dependent neuronal nitric oxide synthase (NOS), and calpains, which are Ca^2+^ activated proteases [42, 43].

In the first few minutes following SCI, oxidative stress, lipid peroxidation, and membranous deposition of protein aggregates take place. These processes impair Ca^2+^ pumps and cell membrane channels, including those present in the endoplasmic reticulum. This downregulation is evidenced by an increased concentration of cytosolic Ca^2+^ from extracellular pools and intracellular Ca^2+^ storages [44]. In normal conditions, the energy-dependent Ca^2+^ buffering system within axons removes the excess Ca^2+^. However, when adenosine triphosphate (ATP) is depleted by the excessive energy demands of demyelination, this normal Ca^2+^ buffering fails and the level of intracellular Ca^2+^ rises until it becomes toxic [44]. The result is the chaotic activation of processes such as proliferation, differentiation, apoptosis, and gene transcription in cells [45].

In addition to the before-mentioned channels, axons also have a high concentration of voltage-gated Na^+^ channels spread along the length of their bodies. Thus, when axonal demyelination occurs, there is a dramatic increase in Na^+^ influx into the cell during the action potential propagation. The elimination of such an excess concentration of intracellular Na^+^ can come at a steep metabolic expense in a similar fashion to Ca^2+^ removal, since the Na^+^/K^+^ ATPase maintains the Na^+^ electrochemical gradient by ATP consumption [46, 47].

When ATP levels fall below a certain threshold, there is a concomitant increase in the intra-axonal concentration of Na^+^ and Ca^2+^. Consequently, glutamate is released, and the Na^+^/Ca^2+^ exchanger, which normally pumped out 1 Ca^2+^ in exchange for 3 Na^+^, is reversed [46, 47].

It is also important to mention that the subsequent release of ATP after the lesion increases in peritraumatic areas for 6 or more hours [48]. This excessive release of ATP by the traumatized tissue after SCI is followed by the activation of high affinity purinergic P2X receptors. It is important to note that the P2X7 receptors may also contribute to the excessive influx of Ca^2+^ since they are upregulated in response to the ATP release induced by SCI. This might explain why spinal cord neurons respond to ATP with excessive firing, followed by irreversible increases in Ca^2+^ that end up in cell death [49, 50].

Furthermore, P2X7Rs have been associated with cells of the immune system that mediate cytotoxic cell death (because of changes in transmembrane ion fluxes, swelling, and vacuolation) and those that mediate inflammatory responses, including proinflammatory mediators such as IL-1 and TNF*α* [49, 50].

### 2.3. Glutamate Excitotoxicity

Glutamate receptors are involved in the excitatory neurotransmission of the mammalian CNS, where they participate in various changes in the efficacy of synaptic transmission, and induce excitotoxic damage in a variety of acute and chronic neurological disorders [51, 52]. The process of excitotoxicity refers to the excessive receptor activation by this excitatory amino acid that results in neuronal death [53].

Just 15 min after SCI, glutamate levels at the epicenter and surrounding regions become six times higher than physiological levels due to the overstimulation of ionotropic receptors and the massive increase of intracellular Ca^2+^ and Na^+^. This glutamate influx provokes overexcitation and endotoxicity by the secondary increase of intracellular Ca^2+^ and the activation Ca^2+^ dependent signaling pathways as previously mentioned [54–56]. Moreover, the augmented expressions of genes related to neurotransmitter receptors (NMDA, AMPA, Ach, GABA, Glur, and Kainate) increase demyelination and oligodendrocyte destruction [57, 58].

An important mechanism for the reduction of excessive extracellular glutamate is the activity of glutamate transporters such as glial glutamate transporter 1 (GLT-1) and glutamate aspartate transporter (GLAST), which are primarily expressed by astrocytes [59]. Unfortunately, the excitotoxicity induced by the extracellular glutamate concentration is enhanced by the reduced uptake by astrocytes and the microglia release TNF*α*, IL-1*β*, and ROS that exacerbated the neural damage [60].

TNF*α* and IL-1*β* have been shown to cause oligodendrocyte death when the latter are placed in coculture with both astrocytes and microglia. Both cytokines inhibit glutamate transporters in astrocytes and thus expose oligodendrocytes to an excessive glutamate concentration. It is important to note that antagonists of AMPA/kainate glutamate receptors such as NBQX (2,3-dioxo-6-nitro-7-sulfamoilbenzo(f)quinoxalina) and CNQX (6-cyano-7-nitroquinoxaline-2,3-dione) blocked IL-1*β* toxicity towards oligodendrocytes [61].

TNF*α* causes excitotoxicity through a series of interconnected, deleterious mechanisms. First, microglia release this cytokine in the inflammatory response, which induces additional release of TNF*α*. In turn, it causes the release of glutamate that acts on metabotropic receptors of microglia and stimulates more TNF*α* release. Subsequently, astrocytes are stimulated to release glutamate, which is not effectively transported back into the soma. Lastly, the rise in the excitatory/inhibitory ratio causes the excessive Ca^2+^ entry and excitotoxic neuronal death previously described. The consequent neuronal death caused by the excessive glutamate concentrations further stimulates microglia to remain in an active state, which includes the production and release of TNF*α* in a vicious cycle [53]. TNF*α* potentiates cytotoxicity by glutamate through an increased localization of glutamate receptors such as AMPA and NMDA while decreasing inhibitory GABA receptors on neurons [62], which explains why NBQX blocked TNF*α* toxicity to oligodendrocytes [61].

### 2.4. Neurofilament Destruction

Spinal cord trauma results in the destruction of neurons, nerve fibers, glial cells, and blood vessels at the site of injury, where approximately 30% of neurofilament constitutive proteins are degraded in 1 h, and 70% are lost within 4 h after the injury [63].

Proteins such as cathepsin B, Y, and S, members of the cysteine lysosomal proteases and papain superfamily, have been linked to neurofilament destruction. This link results from the fact that cathepsin B can degrade myelin basic protein, cathepsin Y can produce a bradykinin, and cathepsin S can degenerate extracellular molecules through inflammatory mediators.

In particular, only cathepsin S is able to retain its activity after prolonged incubation at neutral pH, more than 24 h [64, 65]. The expression of this protease is restricted to cells of the mononuclear phagocytic system such as microglia and macrophages [64]. A basement membrane heparan sulfate proteoglycan (HSPG), perlecan, which was found to promote mitogenesis and angiogenesis, can be degraded by cathepsin S in vitro. HSPGs have roles in adhesion, protease binding sites, and growth factor regulation as is the case for basic fibroblast growth factor (bFGF) [66]. Furthermore, cathepsin S degrades laminin, fibronectin, collagens, and elastin at acidic or neutral pH [65]. It is known that TNF*α*, interferon-*γ* (IFN*γ*), IL-1*α*, and granulocyte macrophage colony stimulating factor (GMCSF) stimulate the release of active cathepsin S into an environment with a neutral pH [65].

Subsequently, a change in lipid metabolism and the homeostasis of lipid mediators is an alternate route by which genes are thought to modulate the susceptibility of nervous tissue to trauma. Interestingly, altered protein cleavage, one of the main driving forces of protein aggregation in neurodegenerative disorders, can be further enhanced by trauma occurring in the presence of specific lipid-binding proteins, important molecules in charge of the distribution of lipids and the transport of cholesterol among cells in the CNS. Apolipoprotein E (ApoE) is one particular example of this phenomenon, since a reduction in its availability causes a reduction in the recovery after neurotrauma or an ischemic insult. ApoE fragments are produced by trauma-induced proteolytic cleavage, which, in turn, might disrupt the cytoskeleton by the phosphorylation of tau and the promotion of neurofibrillary tangles. At the same time, ApoE4 increases the inflammatory effect of neurotrauma by a significant increase of IL-6, TNF*α*, and NO^•^ in the injured tissue [67, 68].

### 2.5. Free Radicals

Microvascular disruption, ionic imbalance, increased intracellular calcium levels, glutamate excitotoxicity, mitochondrial dysfunction, arachidonic acid breakdown, and the activation of iNOS contribute to the formation of free radicals (FR) [69]. FR are reactive molecules produced by the metabolism of the cell that possess an unpaired electron, which easily reacts with biomolecules by oxidizing them [70].

A FR is made up of sulphur (S), nitrogen (N), chloride (Cl), or carbon (C). These elements associate with oxygen and form other FR such as NO^•^. Metals such as Fe, Mn, Co, Ni, and Cu can also be considered FR since they have unpaired electrons [71, 72]. Many of these molecules are either reactive oxygen species (ROS) such as delta and sigma oxygen (O_2_), superoxide anion (O_2_^∙−^), hydroxyl anion (OH^−^), hydrogen peroxide (H_2_O_2_), or reactive nitrogen species [(RNS) NO^•^].

The mechanical reduction of the superoxide anion mediated by NAD(P)H oxidases causes the anion to react with NO and form a neurotoxic compound known as peroxynitrite (O_2_^∙−^ + NO^•^ = ONOO^−^) [73]. At physiologic pH, peroxynitrite first reacts with proteins and phospholipids and then breaks down into other cytotoxic products such as NO^•^, nitrogen dioxide (NO_2_), and OH^−^ radicals.

Hall and Braughler demonstrated the occurrence of early posttraumatic lipid peroxidation (LP) as early as 5 min after injury. LP is a mechanism that disrupts the normal structure and function of the lipid bilayers that surround the cell and membrane-bound organelles. When peroxynitrite or other FR takes an electron off a polyunsaturated lipid, it generates a lipid radical (L^•^) that can further interact with molecular oxygen and yield a lipid peroxyl radical (LOO^•^). Then, if the resulting lipid peroxyl radical LOO^•^ is not reduced by antioxidants, LP associated with SCI induces early damage to the spinal microvascular endothelium (within 2-3 h). As a direct consequence of this damage, there are crater formation, platelet adherence, leucocyte presence, and the formation of microemboli, events that are concurrent with the reduced blood flow to the white matter of the spinal cord. The damage to the myelin sheath unhinged a demyelination process that is the particularity of a neurodegenerative process [74].

The CNS is particularly sensitive to LP because of its high content of peroxidation-susceptible lipids (arachidonic, linoleic, and docosahexaenoic acid) and the primarily radical-mediated oxidative protein damage. Considering the timeframe of the injury, the oxidative damage to DNA and lipids, in addition to protein nitration, is observed within the first week after injury [75–81].

One of the degradation products of peroxynitrite, NO^•^, alters the mitochondrial electron transport chain and induces the production of FR. These molecules have direct deleterious effect on enzymes with iron-sulfur clusters in their catalytic core, such as ubiquinone succinate [82].

After SCI, the concentration of NO^•^ increases 3 to 5 times more than baseline levels and reaches its peak at 12 h. Meanwhile, there is an increased production of inducible nitric oxide synthase (iNOS) and peroxynitrite [83]. The resulting elevated NO^•^ concentration induces cell damage and lipid peroxidation, increases vascular permeability, and causes edema [84]. Hence, due to its involvement in the previous processes, NO^•^ participates in the development of the excessive glutamate and calcium concentrations that induce excitotoxicity [85].

It is known that NO^•^ is produced by different synthases. However, only iNOS is capable of producing a high concentration of NO^•^ for a prolonged period of time [86]. Collectively, astrocytes, neutrophils, monocytes, and microglia induce the expression of iNOS at the presence of proinflammatory stimuli such as lipopolysaccharide (LPS), ultraviolet radiation (UV), and TNF*α*, IL-6, IL-1, and IFN*γ* [87]. In some studies, the expressions of iNOS and its protein activity were found 3 h, 4 h, 24 h, and 72 h after SCI [83, 88, 89].

### 2.6. The Inflammatory Response after SCI

The inflammatory response is a characteristic phenomenon of innate immunity that does not require a previous exposition to the agent but does increase substantially with subsequent expositions as the response becomes specific and direct. Cellular immunity consists of specialized cells that can recognize, endocyte, and eliminate different types of microorganisms or noxious substances. On the other hand, the humoral response is composed by soluble macromolecules that circulate in the blood and extracellular liquid that acts upon the pathogen [90, 91].

SCI presents different patterns of gene expression depending on the cell type and activation phase [92]. Numerous studies have suggested that the inflammatory response in SCI is beneficial, because it can eliminate tissue debris and induce the release of various neurotrophic factors [17, 93, 94]. Nevertheless, this inflammatory response tends to go out of control when it exacerbates autoreactive mechanisms that cause neural destruction. Traumatic SCI triggers inflammatory reactions in which various types of cells, cytokines, and neuroprotective/neuroregenerative molecules are involved [95].

#### 2.6.1. Cells of the Inflammatory Response

Immediately after the rupture of the blood-spinal cord barrier, the consequent inflammatory response involves the participation of chemical mediators, and resident (astrocytes and microglia) and peripheral (macrophages, lymphocytes) immune cells [96, 97]. Additionally, oligodendrocytes, neurons, and endothelial cells participate in the cellular response after SCI [98], in which microglia and endothelial cells function as antigen-presenting cells (APC) [96].

Throughout the inflammatory response, the infiltration of immune cells is the principal contributor to neural degeneration [95]. These cells are guided to the lesion site from the periphery by the effect of chemokines and cytokines that are mainly released by microglia, astrocytes, and peripheral macrophages, which make up the principal resource of these molecules in the lesion site [5, 99–102]. The released cytokines include IL-1*α*, IL-1*β*, IL-6, TNF*α*, GM-CSF, and LIF [60]. The neurons of human patients expressed these molecules, 30 min after SCI, and microglia, 5 h after the lesion; however, the expression decreased by the 2nd day [103]. Similar results were obtained in mice and rats since the expression by local neurons was found at 1 h, and at 6 h by microglia, which decreased to baseline on day 1 after SCI [104]. The expression of TNF*α* and IL-1*β* by microglia and astrocytes was identified 5–15 min after the lesion. The peak expression was at 1 h for TNF*α* and 12 h for IL-1*β* [104].

After SCI, two waves of cellular infiltration have been characterized. The first wave consists of polymorphonuclear leukocytes (PMN) that predominate throughout the first hours following the lesion. They are activated by IL-1, interleukin-2 (IL-2), and IL-6 in particular [105] and might be mainly recruited by chemokine (C-C motif) ligand 2 (CCL2), chemokine (C-X-C motif) ligand 1 (CXCL1), and chemokine (C-X-C motif) ligand 2 (CXCL2, also called macrophage inflammatory protein 2-alpha (MIP2-alpha)) [106]. These cells become apparent in the walls of veins and venules adjacent to the lesion in the first 3-4 h and can be observed inside the tissue 8–24 h after the lesion. It has been found that these cells represent 90% of the infiltrating cells 12 h after the injury [107].

The inflammatory response is evidenced by the increased quantity of leukocytes in the cerebrospinal fluid, the infiltration of PMN in the lesion site, the increment in the leukotriene levels (LTB4 in particular), and the activity of myeloperoxidase. In addition, a significant increase in the expression of intercellular adhesion molecule 1 (ICAM-1) can be identified, which favors the infiltration of neutrophils from the first 3 to 12 h after the lesion [108].

The second wave of infiltration is characterized by the presence of monocytes and macrophages, which can be observed around 3-4 days after SCI [106]. Various chemokines are known to mediate macrophage infiltration such as CCL2, CXCL1, and CXCL2 [106]. This demonstrates how important the recruitment of macrophages is after an injury to the CNS [109, 110]. Activated microglia become evident in the first day after SCI [108]; moreover, there is a peak in the proliferation and recruitment of microglia from day 3 to day 7 [111, 112]. The overexpression of LIF has been found to cause a dramatic increase in the proliferation of microglia/macrophages and astrocyte activation [24]. The pathological proliferation of macrophages and microglia might contribute to the subsequent exacerbation of the initial damage [113, 114], even though macrophages have an important role in the clearing of denatured dendrites [115]. Microglia at the injury site rapidly express the alarmin IL-1*α*, while infiltrating neutrophils and macrophages produce IL-1*β* which plays a role in the infiltrating mechanism of these cells. Interestingly, the expression of IL-1*α* mediates the suppression of the survival factor Tox3 (TOX High Mobility Group Box Family Member 3) in oligodendrocytes, which in the absence of such cytokine would provide protection of this cell population, and functional recovery after SCI [7].

Diverse studies have reported that the recruitment of leukocytes to the injured spinal cord is a physiological response that is associated with the production of cytokines and protein kinases that are involved in the repair of the site of lesion. Neutrophils, for example, are the first cells to be recruited with the objective of clearing the lesion site from possible pathogens and cellular debris through phagocytosis. However, the activation of these cells also induces the release of a significant amount of neurotoxins such as ROS, RNS, chemokines, and a variety of enzymes that promote tissular destruction [105, 116, 117]. The Taoka report provides evidence demonstrating that after SCI the maximum peak of neutrophil migration perfectly correlates with the extent of the damage and the motor alterations observed after the lesion [105].

The infiltration of monocytes and macrophages after SCI has for its objective the removal of cellular debris and the stimulation of new blood vessel and parenchymal cell formation. The infiltration of these cells regulates the activation and proliferation of T lymphocytes since they play the role of APC [117].

Microglia are pluripotent cells capable of developing different phenotypes proportional to the severity of the lesion, which determines the intensity of the inflammatory response, the quantity of recruited cells, and the magnitude of the immunological response. This can be explained by the interaction between microglia and T lymphocytes, since it induces an antigen specificity that regulates the immune response and the subsequent phases [118]. Microglial cells are distributed throughout the CNS, where they serve as a pathological sensor that is activated in response to noxious stimuli such as physical trauma or vascular obstruction [119]. Activated microglia migrate to the site of injury, proliferate, and transform from the resting phenotype (ramified cells) to amoeboid phagocytic cells [120]. In fact, after SCI, activated microglia can be seen at the epicenter of the lesion initially at 12 h [60]. In addition, there is an upregulation of surface receptors such as CR3 (complement receptor type 3) and MHC II (major histocompatibility complex) whose implications are covered in several papers of our group. These activated microglia can then release a series of cytokines, chemokines, and enzymes, which are proportional to the activating stimulus as mentioned previously. The series encompass IL-1*β*, IL-6, TNF*α*, TGF-*β*1 (transformation growth factor-*β*1), M-CSF [121], iNOS, NGF (neural growth factor), NT-3 (neurotrophin-3), and BNDF (brain neuronal derived factor) [122, 123]. Interestingly, monocyte derived microglia and macrophages might be able to induce regeneration by the secretion of neurotrophic factors, particularly TGF-*β*1 [17]. Activated microglia and macrophages have been implicated in the secondary pathology that accompanies traumatic or autoimmune injuries to the brain and spinal cord [124]. The associated implications usually refer to the activation of these cells towards an inflammatory M1 phenotype; however, these cells can also be activated towards an M2 macrophage phenotype that responds to IL-4 and IL-13. This contrasting phenotype is characterized by the production of several extracellular matrix proteins that may promote tissue remodeling, repair, neurotrophic support, and axonal regeneration [125–128].

Taking into account the excessive release of glutamate and the feedback of the inflammatory response after SCI, it is no surprise that microglia acquire a reactive phenotype that expresses very low quantities of the MHC II molecules and is not capable of maintaining an adequate interaction with T lymphocytes [118, 129]. The M1 phenotype is characterized by the excessive release of NO^•^, IL-1, IL-6, and TNF*α*, which leads to toxicity. In these conditions, astrocytes and postsynaptic neurons show signs of damage, evidenced by the expression of ROS, which can induce apoptosis [121]. Nevertheless, activated microglia remove the cellular debris after the lesion and are capable of promoting revascularization in the site of injury, which facilitates the release of trophic factors and nutrients for the survival and proliferation of infiltrating cells in the lesion site [118]. Furthermore, microglia are capable of expressing glutamate transporters, which apparently help buffer the excessive concentrations of glutamate, and consequently protect cells from toxicity [129, 130]. It is important to take the before-mentioned into account since activated microglia and peripheral macrophages make up the majority of inflammatory cells present in the site of lesion, especially since these cells are morphologically different and respond to different modulatory signals [131] in an early response of the innate immunity to the lesions of the CNS, which have diverse etiologies such as ischemia and neurotrauma [132, 133]. Therefore, given that in the brain and the spinal cord there is a considerable heterogeneity of macrophages, the relative contribution of one population of cells in the local inflammatory reaction can dictate whether a cascade of events initiates as a regenerative or a destructive process. This all depends on the macrophage phenotype activated, particularly microglia [117].

In regard to T-lymphocyte infiltration in humans, it might be detected months after the injury, and B-lymphocytes are not usually found [134]. On the other hand, mice models present both cell types 7 days after SCI, with a peak at 42 days [135]. Lymphocytes are the cells that modulate the intensity of the inflammatory response. Traditionally, their participation after SCI has been linked with the damage to neural tissue since they are capable of producing proinflammatory cytokines such as INF*γ* and IL-1*β*. On one hand, INF*γ* is linked directly to neuronal destruction since it induces the expression of other proinflammatory cytokines (TNF*α*, IL-6, IL-12, and IL-1*β*), and proinflammatory molecules such as ROS and iNOS, since it participates in the induction of transcription factors including NF*κβ* (nuclear factor kappa beta) and AP-1 (activator protein-1) [118, 136, 137].

In addition, INF*γ* is known to participate in the induction of a cytolytic response by TCD8+ (CD, cluster of differentiation) since it is the principal inductor of MHCI through the phosphorylation of STAT1 (signal transducers and activators of transcription-1) [138]. Moreover, the chemoattractant, C-X-C motif chemokine 10 (CXCL10) recruits CD4 Th1 cells via the CXCR3A receptor [95].

After the induction of protective autoreactivity, which is a strategy based on the modulation of the immune response by neural derived peptides, diverse studies have reported that the presence of T lymphocytes with a Th2 phenotype in the lesion site favors functional recovery [139–141]. This is due to their ability to synthesize NGF, BDNF, and diverse neurotrophins (NT3, NT4, and NT5) [142, 143].

In a classical Th1 activation pattern, however, the inflammatory response after SCI can be responsible for the necrosis of the lesion site and the surrounding area [144–146]. To further explore this phenomenon the cytokine expression was analyzed in comparison to sham animals and the dominant phenotype was found to be Th1 and Th17 predominantly according to expectations [147, 148]. This is due to its important role in the generation of free radicals, cytokines, and chemokines that exacerbate the damage to the neural tissue for weeks or even months. It is important to point out that this noxious effect occurs when the immune response is not modulated, since it is correlated with excitotoxicity, lipid peroxidation, and the development of an autoreactive response towards neural constituents [17, 93, 139, 149, 150]. Contrastingly, this response can either increase the damage to the neural tissue, promote protection [150–153], or even promote restoration of the injured tissue [126, 127, 144] as a function of neuromodulation.

The autoreactivity observed after SCI is characterized by the specific immune response, with lymphocytes being the only cells capable of specifically recognizing the antigens, and initiating the adaptive immune response. Despite the existence of mechanisms by which these autoreactive T cells are eliminated or inactivated, these are not sufficient, and consequently they can be found in practically every healthy individual. Thus, autoreactivity can be part of a normal immune response that can find its origin in several infectious and inflammatory diseases. However, when this mechanism is excessive, the result is an autoimmune disease [154–156]. There are multiple diseases that are considered to be autoimmune or to have an autoimmune component. Multiple sclerosis (MS) is one of such diseases. It is an inflammatory, demyelinating disease, in which an autoimmune response to MBP [157] has been reported. Interestingly, after SCI, an autoreactive phenomenon similar to the pathophysiology of MS can be observed. Consequently, it is well-known that the lymphocyte role after SCI is fundamental, because these cells are responsible for the generation of autoimmunity in individuals with genetic susceptibility [89, 158, 159].

These events can become chronic if the proinflammatory environment is not regulated. If not regulated, the response would involve the participation of other immune cells, other signaling pathways, and other patterns of gene expression. The persistent influx of immune cells from the systemic circulation as neutrophils, macrophages, lymphocytes, basophils, and eosinophils is correlated with additional elevation of proinflammatory cytokine levels and neural tissue destruction that would unavoidably make tissue recovery more difficult [108, 160, 161].

#### 2.6.2. Cytokines of the Inflammatory Response

Cytokines comprise a large family of small signaling proteins that affect nearly every biological process including embryonic development, disease, nonspecific infection response, cognitive functions, aging, cellular growth, survival, and differentiation [10, 162]. These “cytokines,” which can be classified as peptides, proteins, or glycoproteins, encompass interferons, interleukins, the chemokine family, the tumor necrosis factor family, adipokines, and mesenchymal growth factors [10, 163]. These molecules are produced by one cell and go on to act on another cell in order to bring a change in the function of the target cell. The difference with hormones is that cytokines are products of most cells while not being of a particular tissue or cell. The majority of cytokines function by binding to specific cell surface receptors; this action triggers intracellular signaling and activates transcription factors such as AP-1 and NF*κβ* [162]. Interestingly, the diverse properties of a single cytokine can be explained by the following mechanisms: the first mechanism involves the presence of the receptor of a certain cytokine in one particular type of cell (e.g., IL-33 receptor on mast cells) [164]. The second mechanism is explained by the presence of the receptor to a specific cytokine on most cells (e.g., activation of NF*κβ* by IL-1, or TNF*α* induction of COX-2). The third mechanism encompasses the ability of cytokines to induce or function as coactivators (e.g., IL-18 induces IFN*γ* when IL-12 is present, but when it is not, IL-18 induces Fas ligand) [165]. Despite the fact that cytokines are studied in every discipline of biology, the effects of these molecules are mostly studied in the realm of inflammation, immunology, cancer, and atherosclerosis [162]. In these areas, cytokines can be grouped into a proinflammatory or anti-inflammatory category on the basis of the resulting balance of their added effects [10].

In the CNS, cytokines have homeostatic physiologic and neuromodulatory functions. Surprisingly, they also have the capability of contributing to neuronal damage and destruction when their concentration exceeds a certain threshold. One of the reasons as to why they cause such damage and destruction lies in the uncontrolled inflammatory response observed after SCI, which emphasizes the reason behind the augmented study of these molecules in inflammation-related research. The upregulation of these cytokines, as well as the consequent cellular infiltration they cause, plays a crucial role in the determination of the extent of the secondary tissue damage and neural degeneration observed after the injury [95, 166, 167].

Therefore, taking into account that the production and release of proinflammatory cytokines and chemokines (Table 1) is the first inflammatory event that develops after SCI, the importance of these molecules becomes clear [166, 167]. In regard to the realm of inflammatory cytokines, there is a clear diversity in their functions. For starters, certain molecules are capable of inducing vascular permeability and cellular fluid loss, which include components of the complement cascade (C3a and C5a), which in turn cause the release of histamine, prostaglandins, and leukotrienes from resident mast cells. Specific inflammatory cytokines such as TNF*α*, IL-1, and IL-6 are synthetized by various cells in the CNS and are known as mediators of the peripheral immune response [168–170]. On one hand, TNF*α* immediately recruits neutrophils to the site of the lesion by the induction of adhesion molecules such as ICAM-1 and VCAM-1 (vascular cell adhesion molecule-1), as it stimulates the release of IL-8, which is a chemotactic factor for neutrophils. Furthermore, TNF*α* alters the permeability of endothelial cells and damages the blood-spinal cord barrier. Moreover, this cytokine is able to exert cytotoxic activity towards oligodendrocytes and contributes to demyelination. In addition, TNF*α* also stimulates the proliferation and hypertrophy of astrocytes, hereby promoting the formation of the fibroglial scar, which acts as a barrier to a possible regeneration of the CNS as a biological measure of last resort in response to an uncontrolled chronic inflammation [168–170]. On the other hand, studies have shown that a direct injection of IL-1 into the spinal cord leads to enhanced vascular permeability and lymphocyte recruitment. Subsequently, IL-6 has been found to promote the activation and infiltration of macrophages and microglia [161, 171]. In fact, it is known that IL-6 is a major player in chemokine infiltration, because it has the ability to interact with other cytokines and neurotrophic factors [172, 173]. Interestingly, several studies have revealed that the continuous inhibition of IL-6 is detrimental to functional recovery because it also participates in axonal regeneration and gliosis, in line with the role of TNF*α* in chronic inflammation [174, 175]. Thus, it is important to take into account that the mediation of the early inflammatory tissue damage may actually worsen the functional outcome [176].

This leads to a conflict, since the role of inflammation after SCI appears to be contradictory when the before-mentioned and following points are taken into account [177]. On one hand, proinflammatory cytokines, IL-1*β* and IL-6, are beneficial at low concentrations due to their induction of neurotrophin expression and the mediation of leukocyte activation/recruitment to the injury site by the induction of adhesion molecules in the cell surface such as ICAM-1, P-selectin, and E-selectin [172, 173]. On the other hand, at higher concentrations, these inflammatory cytokines activate transcription factors such as NF*κβ*, AP1, and ATF, factors that stimulate the expression of neurotoxic genes, including COX-2, iNOS, and proinflammatory proteases in different target cells [88, 178, 179].

Pan found that the mRNAs of cytokines such as TNF*α*, IL-1*α*, IL-1*β*, and IL-6 could be detected 15 min after injury. From these cytokines, IL-1*α* and IL-1*β* continually reached peak levels until the 6 h but were not present from the 12 to 24 h after SCI. In addition, by 4 h after contusive SCI, significantly increased mRNA levels of IL-1a and IL-6 were clearly detected by qRT-PCR [180, 181]. Digging further into the time frame of expression, western blot studies found that the mature form of IL-1*β* is expressed by the 2 h. This evidence suggests that the inflammatory cytokine is released very quickly after tissue damage. The expression of these genes was identified 1 h after contusive rat SCI by cDNA microarrays [57]. The procedure was then repeated in spinal cord injury patients, and the same results were observed [103]. Moreover, Hayashi found that after SCI the mRNAs of cytokines such as TNF*α* and IL-1 were upregulated in as little as 1–3 h after the lesion [148, 182, 183]. On another note, TNF*α* mRNA peaked quickly 60 min after the injury and fell slightly by the 120 min. TNF*α* mRNA remained elevated by day 1 after SCI, returned to a low level by day 3, and was not detected by day 5 [184]. IL-6 mRNA increased slowly, reached peak levels by 6–12 h, and fell by 24 h [180]. It is important to note that the levels of these mRNAs were nearly undetectable in sham-injured animals. Another study found that, between 12 h and 72 h after SCI, the gene expression of proinflammatory cytokines such as IL-1, IL-3, IL-6, and their receptors was strongly upregulated [6].

TNF*α* and IL-1 induce both IL-1 and TNF*α* mRNAs. Consequently, the downregulation of the signaling of IL-1 and TNF*α* reduces the induction of IL-1*β* mRNA [163]. This suggests that the activity of these cytokines contributes to their own mRNA regulation [163, 180]. From the 3 h and up to 24 h, TNF*α*, IL-1*β*, IL-6, and LIF were found to be strongly upregulated in and around the contused area. These cytokines were produced at the same time range. It is worth noting that another wave of expression was observed for TNF*α* and IL-1*β* at 14 days, which correlates with an increased blood-spinal cord barrier function [104]. In particular, the overexpression of LIF has been found to cause a dramatic increase in the proliferation of microglia/macrophages and astrocytic activation [24].

TNF*α* is released significantly faster than other proinflammatory cytokines, because this is stored in a preformed state on the cell surface and in the granules of mast cells. It is not a surprise that role of this cytokine is similar to that of IL-1*β* given the facts stated above [185]. It is important to note that TNF*α* is the principal promoter of Wallerian degeneration since it activates resident Schwann cells in the peripheral nervous system and facilitates macrophage recruitment into the injury site [186]. In addition, these macrophages release proteases, FR, and cytokines [187]. Similar to the facts stated above, the extracellular expression of TNF*α* [187] in the surrounding white matter was detected 3 h posterior to contusion SCI, with a peak that took place from day 1 to day 3 [166].

Thus far, the time frames of expression have been described. The following information regards the receptors of such molecular products. From the two subtypes of TNF receptor that exist, each subtype has a different distribution and presence that depends on the particular cell type. For instance, TNF-R1 is expressed constitutively on most cell types, whereas the expression of TNF-R2 in astrocytes requires induction by TNF*α*, IL-1*β*, and IFN*γ* [188]. A large amount of evidence indicates that TNF-R1 augments neuronal death and TNF-R2 promotes neuroprotection [189]. What has been observed in the lesion concludes that the expression of TNF-R1 and TNF-R2 is increased within 15 min after traumatic SCI in adult rats and reaches its peak at 4 h for TNF-R2 and 8 h for TNF-R1.

The expression of both receptor subtypes then goes on to decline after day 1 and day 3, respectively [190]. It is important to note that these receptors are initially found on the epicenter of the lesion site. Posteriorly, they spread radially towards distant areas during their peak expression and later become confined to the lesion area. These receptors are expressed by several cells, which include neurons, oligodendrocytes, and astrocytes [189, 190]. These cells might work individually or synergistically to mediate the biological activity of TNF*α*, which makes an interesting research topic, given that these receptors are known to be involved in antiapoptotic activities through the TNF-R/NF*κβ* signal transduction pathway [191]. On a last note, TNF*α* participation in the expression of iNOS in microglial cells [137] causes an exacerbated neural destruction as a direct consequence of the induction of the NF*κβ* pathway, which can then contribute to the expression of IFN*γ*.

IFN*γ* within the nervous system is classically associated with the inflammatory response after injury as mentioned in the previous paragraph [213]. This molecule is believed to be normally involved as one component of the physiological response to tissue damage and trauma. CD4+ and CD8+ T cells together with natural killer (NK) cells are the major sources of IFN*γ*. Nevertheless, evidence shows that this cytokine is also produced within the nervous system by neurons and glial cells in the absence of infiltrating immune cells [214]. In various animal models, IFN*γ* promotes macrophage signaling, production of proinflammatory cytokines and chemokines, recruitment of macrophages to the CNS, and the activation of CNS resident and infiltrating APC populations. Moreover, IFN*γ* is also the most potent inductor of MHCI, and it is upregulated in the CNS after injury [215]. In low concentrations, IFN*γ* may participate in the homeostasis of the synaptic circuitry [216, 217]. As previously mentioned, IFN*γ* is involved in the upregulation of MHC I, which has been shown to play an important role in the synaptic plasticity process following axotomy. Furthermore, IFN*γ* has been shown to regulate phosphorylation and nuclear translocation of signal transducer and activator of transcription 1 (STAT1) and to influence neuronal excitability by the expression of the peripheral nerve-type sodium channel gene PN1 [192]. It is important to note that several studies found that IFN*γ* and IL-17 had the highest levels of gene expression, since this indicates that the phenotype found after SCI is predominantly Th1 and Th17 and the IFN*γ* release could be detected from 1 h to 12 weeks, depending on microenvironment [147, 148].

Interleukin-17 (IL-17) is primarily produced by Th17 cells and has an important role in inflammation and autoimmune disease [201]. A key regulator in its production is IL-6. Nevertheless, TGF-*β* and interleukin-21 (IL-21) are also capable of stimulating IL-17 production. Similarly, interleukin-23 (IL-23) is also able to promote IL-17 production just as interleukin-22 (IL-22) does. In one study, serum levels of IL-6, IL-21, and IL-23 were increased in large quantities 1 h after SCI, had a peak at 24 h, and had a positive correlation with increased IL-17 [202]. Signal transducer and activator of transcription 3 (STAT3) and RAR-related orphan receptor gamma (ROR*γ*) are two transcription factors capable of mediating IL-17 production and Th17 differentiation. As a result, a closed circuit is formed, in which IL-17, the STAT3 signaling pathway, and IL-17 related cytokines promote neuroinflammation as they costimulate one another. IL-17 expression and production was detected from 1 h to 72 h after contusion injury [202].

STAT3 is a primary transcription factor of the downstream signaling of IL-6 [203]. The phosphorylation of STAT3 in this pathway induces a proinflammatory gene expression that correlates with IL-17 quantities in spinal cord neurons and astrocytes. Interestingly, through this very same pathway, anti-inflammatory Th2 cells can be suppressed by IL-6 inhibition of Foxp3 expression in a STAT3 dependant manner. It is important to recognize that STAT3 was found higher in the SCI rat group, whose expression peaked at 24 h [202].

It is worth noting that IL-17 antagonistic therapy in rheumatoid arthritis (RA) suggests that the inhibition of the pathological role of IL-17 may be a promising therapeutic approach in humans [204].

#### 2.6.3. Chemokines of the Inflammatory Response

Chemokines are functionally related cytokines that induce specific actions in the immune system. They are released in response to an infection, inflammation, or trauma [184]. Chemokines are grouped into two families: the *α* family (CXC), which participates in the recruitment of polymorphonuclear cells, and the *β* family (C-C), which provides the priming signal for macrophages, lymphocytes, eosinophils, and basophils.

The *α* family includes gamma-interferon inducible protein (IP-10/CXCL10), platelet factor 4, IL-1, and melanoma growth stimulatory activity (MGSA/gro/KC) [218]. In particular, the chemokine CXCL10 has been shown to inhibit angiogenesis, growth, and chemotaxis of endothelial cells via the CXCR3B receptor. Consequently, the neutralization of CXCL10 promotes angiogenesis through the expression of eight genes related to angiogenesis and vasculature remodeling after SCI [95].

An important member of the *β* family is the monocyte chemoattractant protein (MCP-1/CCL2). It is detected in astrocytes and perivascular mononuclear cells in experimental allergic encephalomyelitis (EAE). MCP-1 levels are related to the parallel development of clinical disease and macrophage infiltration [205, 206]. The same case applies to macrophage inflammatory protein 1 alpha (MIP-1*α*/CCL4) and macrophage inflammatory protein 1 beta (MIP-1*β*) [219]. Their expression has been shown predominantly in myeloid and lymphoid cells [207], where an increased expression of MIP-1, MIP-2 (CXCL2/3), and MCP-1 after SCI plays a role in the inflammatory process, since these molecules recruit circulating leukocytes to the injury site [220].

MCP-1 mRNA was present in the normal spinal cord, was increased 1 h after SCI, peaked at 24 h, and returned to a low level by day 14. MCP-1 is expressed by astrocytes that surround white matter. In addition, MIP-1*α* mRNA was present in the normal spinal cord, where it increased at 1 h after SCI, peaked from 3 to 6 h, decreased by day 1, remained unchanged until day 7, and returned to a low level by day 14. MIP-1*β* expression in astrocytes was observed from day 3 to day 6 following injury. Additionally, the expression of this molecule was found at the contusion site and in rostral and caudal sections to this location. By day 5 after injury, the expression of MIP-1*β* returned to baseline levels. Moreover, IP-10 mRNA presented low levels in the normal spinal cord, increased its levels at 1 h, peaked at 6 h, and remained high up to day 5 after SCI. It decreased to baseline levels by day 14 [184].

Another study found the chemokines, MCP-1, MIP-1, MIP-1*α*, MIP-2, and IP-10, to be expressed locally at 30 min with a peak at 6 h after SCI. It is worth noting then that chemokines remain present 24 d after injury—at lower levels—in contrast with the rest of the cytokines [200].

#### 2.6.4. Neuroprotective and Neuroregenerative Molecules of the Inflammatory Response

The changes in gene expression that contribute to the secondary injury are characterized by protracted neuronal loss and neurological dysfunction. Therefore, the predominant downregulation of these factors might play a role in cell survival and may lead to the development of novel interventions that promote recovery [181, 221, 222]. In order to develop a viable therapy, it is essential to identify the specific molecular pathways that become altered as a function of time after SCI [223]. For instance, activated macrophages and microglia after CNS injury produce various neurotrophic factors and molecules that enhance regeneration [93, 224]. However, this response highly depends on the temporal sequence that proceeds the injury [108]. This consequently indicates that there is a proper and timely regulation of inflammatory reactions that can take place and be of paramount importance to the design of therapeutic strategies involving cytokines, growth factors, or neurotrophins [98, 116].

*(1) Cytokines*. A particular cytokine involved in this beneficial aspect of the inflammatory response is IL-4. This cytokine exerts an anti-inflammatory effect after CNS damage [193–195]. For instance, endogenous IL-4 has been shown to participate in the regulation of neuroinflammation in various pathologic conditions [196–198]. This anti-inflammatory cytokine and its receptor subunit IL-4*α* have a role in spinal cord trauma. This is illustrated by the high level expression of IL-4 24 h after contusive SCI in rats, whose elevated concentration persisted for 7 days but was decreased 3 days after SCI. Interestingly, on day 1 after SCI, an increased expression of IL-13 was observed. This is noteworthy since this interleukin shares the same receptor with IL-4 for signal transduction [166, 199]. Moreover, the cytokine expression of the contused spinal cord was not significantly affected by IL-4 attenuation for the proinflammatory cytokine levels of IL-1, IL-6, and TNF*α*. In fact, the opposite effect was observed, since the event correlated with a marked increase in the extent of macrophage quantity 7 days after SCI, which was preceded by an increase in the level of MCP-1 [166]. These results suggest that the expression of IL-4 regulates the extent of macrophage activation in the acute phase of the injury [166]. In addition, IL-4 has been shown to exert a neuroprotective effect against microglia-mediated neuronal toxicity by the regulation of FR formation [194]. On similar lines, macrophages stimulated with IL-4 are reported to be less neurotoxic and to have an increased regenerative capability. This evidence makes IL-4 injections a possible therapeutic application [166].

IL-10 and TGF*α* have been reported to act as neuroprotective molecules in a manner similar to IL-4 [225]. For instance, it has been shown that an intrathecal infusion of TGF*α* is able to enhance axonal growth after spinal contusion through the epidermal growth factor receptor (EGFR) that is primarily upregulated by astrocytes surrounding the lesion. Here, TGF*α* stimulates proliferation, migration, and transformation to an axon phenotype supportive of growth [226]. On the other hand, a potential treatment for certain aspects of the secondary injury such as inflammation, excitotoxic damage, and neuronal apoptosis is the administration of IL-10 since its anti-inflammatory effects involve the downregulation of IL-1*β*, IL-2, IL-6, TNF*α*, IFN*γ*, matrix metalloproteinase-9, nitric oxide synthase, myeloperoxidase, and ROS [227]. In addition, proapoptotic factors such as cytochrome c, Bax, and caspase 3 are downregulated by the effects of IL-10. Other effects of this cytokine include the upregulation of antiapoptotic factors such as B-cell lymphoma 2 (Bcl-2). Furthermore, IL-10 provides trophic support to neurons by its receptor, in addition to increased tissue sparing, neuroprotection, and functional recovery. In the nervous system, IL-10 receptor expression has been found in microglia, astrocytes, and oligodendrocytes acting as antagonist for the production of proinflammatory cytokines [225, 227].

In the first moments after SCI, the elevated synthesis and release of proinflammatory mediators plays a role in the secondary degeneration [103]. This might be a therapeutic opportunity. For instance, an antagonist of proinflammatory cytokines such as IL-1 receptor antagonist has demonstrated a neuroprotective effect after global ischemia, excitotoxicity, and traumatic brain injury in rodents [228].

*(2) Growth Factors*. After mechanical trauma, astrocytes and neurons release fibroblast growth factor (Fgf) which is thought to counteract excitotoxic or ischemic damage by the activation of antiapoptotic signals in stressed neurons [229].

Acidic fibroblast growth factor (aFGF) is a potent mitogenic and chemotactic agent for vascular endothelial cells, dermal fibroblasts, and epidermal keratinocytes. Moreover, it has a role in the regeneration process since it contributes to angiogenesis. In the normal uninjured spinal cord, aFGF mRNA was found to be present in low levels. After SCI (Table 2), however, the factor increased in the 1 h, stayed at that level, peaked from day 5 to day 7, and remained high from day 14 to day 21 [209].

Many therapeutic strategies seek to induce a higher expression of neurotrophic factors. A particular strategy that has shown significant results is the combination of peripheral nerve grafts with aFGF after transection SCI in rats. This strategy induced higher IL-4, IL-10, and IL-13 levels in the graft areas of rat spinal cords. Moreover, this strategy has been shown to regulate Th2 cytokine production, M2 response, and neurotrophic factor production, where the latter can indirectly regulate the inflammatory response and neural destruction [211].

It is worth noting that the use of aFGF with fibrin glue in combination with surgical neurolysis for nonacute SCI has been proven feasible and safe in clinical trials which have shown significant improvements in ASIA motor and sensory scale scores and impairment scales, neurological levels, and functional independence measures, 24 months after treatment [230].

After the injury, the expression of basic fibroblast growth factor (bFGF), another growth factor involved in angiogenesis, was found in astrocytes localized at the site of contusion and in the surrounding white matter. Unlike aFGF, bFGF mRNA was not detected in the uninjured spinal cord. It was only detected 1 h after SCI, in increased quantities at 6 h, and at its peak 3 days after SCI. Afterwards, it remained high from day 5 to day 7, only to return to a low level by days 14 to 21 [209].

Before going further, it is important to note that growth factors such as TGF-*β* may act as immunosuppressants. Moving on, 24 h after SCI, genes related to growth and differentiation became present. These included TGF-*β*, nerve growth factor (VGF), platelet derived growth factor (PDGF-*α*), galanin, and neuropeptide Y. These genes have been suggested as aids in tissue stabilization, structural preservation, repair, and regeneration after SCI. For instance, increased PDGF and VGF levels after SCI may prevent the death of axotomized neurons and a decrease in their energy metabolism [212].

Subsequently, the increased abundance of galanin and neuropeptide-Y transcripts may produce an antinociceptive effect in the injured spinal cord [231]. Moreover, it is known that cannabinoid receptor 1 (CB1) is colocalized with the neuropeptide CCK. In this relationship, the neuropeptide acts as an endogenous opioid antagonist [232]. Therefore, the downregulation of CB1 and the expression of the CCK precursor might help explain why there is a relative resistance of neuropathic pain to the analgesic action of morphine in SCI patients [233]. Similar results have been found in several transcripts, and the previously mentioned genes have shown an increased abundance in comparison to sham animals [57, 223, 234].

*(3) Neurotrophins*. Neurotrophins constitute a family of molecules that has assumed a central role in studies dealing with recovery after SCI [235]. Four members of this family are involved in neuron survival and the regeneration process after SCI: NGF, brain derived neurotrophic factor (BDNF), neurotrophin-3 (NT-3), and NT-4/5. Neurotrophins emit signals when they bind to low and high affinity receptors in the membrane of their target cells. For instance, the low affinity p75 receptor binds all neurotrophins [208]. Another signaling method used by neurotrophins is carried out by three high affinity tyrosine kinase receptors, collectively known as trk receptors. TrkA, TrkB, and TrkC compose the trk family of tyrosine-protein kinases. These three receptors mediate the biological properties of the NGF family of neurotrophins. TrkA is the particular receptor for NGF, while TrkB serves as a receptor for both BDNF and NT-4. Lastly, TrkC is the primary receptor for NT-3. However, this particular neurotrophin can activate TrkA and TrkB receptors when present in high concentrations [236]. Through semiquantitative RT-PCR in a spinal cord contusion model, it was found that the expression of neurotrophin family members and their receptors was significantly diminished 6 h after the lesion. Yet, in contrast to this pattern of Trk receptor expression, p75NTR showed a significant upregulation after contusive SCI [237]. Interestingly, an increase in BNDF was observed up to 6 weeks after compression SCI with a decrease 12 weeks afterwards [210]. Similarly, an increased expression of growth, angiogenic, and axonal guidance factors, as well as extracellular matrix molecules, can be observed in the chronic phase (days to years) following SCI [150, 209].

## 3. Concluding Remarks

The series of interconnected deleterious mechanisms of the secondary injury is orchestrated by the expression of specific genes, in particular those of signaling proteins such as cytokines, chemokines, and growth factors. The balance between the proinflammatory and anti-inflammatory effects of these molecules plays an important role in the progression and outcome of the degenerative process. Most of these cytokines have a dual role in a range between beneficial and injurious, depending on time and the cell implicated in secondary injury after SCI. The excessive and uncontrolled inflammatory response after SCI enhances the damage role of these cytokines, which surpasses the regenerative effects of anti-inflammatory cytokines and growth factor. Consequently, therapies that focus on promoting the anti-inflammatory properties of cytokines and growth factors should be a priority.

## Figures and Tables

**Table 1:** Cytokines and chemokines after SCI.

Item	Detection timeframe	Roles	Reference
IL-1*α*	(i) Early 15 min, PL at 6 h AI	(i) Important alarmin that induces inflammatory response (ii) Enhances vascular permeability (iii) Augments lymphocyte recruitment	[7, 88, 163]

IL-1*β*	(i) Its production starts from 3 to 24 h, PL 12 h AI (ii) Upregulation at 14 days	(i) Astrocytic glutamate transporter inhibitor (ii) Vascular permeability enhancer and lymphocyte recruiter (iii) Low concentrations: (a) Induces adhesion molecules (b) Increases neurotrophin expression (c) Mediates leukocyte activation/recruitment (iv) High concentrations activate the expression of NF*κβ*, AP1, ATF, COX-2, iNOS, and proinflammatory proteases in astrocytes, neutrophils, monocytes, and microglia	[104, 163]

IL-6	(i) Early production at 15 min AI and could be found up to 3–24 h AI (ii) Detected serum levels 1 h AI, PL 24 h	(i) Activates PMN, macrophages, and microglia (ii) Induces the following: (a) iNOS enzyme in astrocytes, neutrophils, monocytes, and microglia (b) IL-17 production (iii) IL1*β*-like effects	[118, 136, 137]

TNF*α*	(i) Early production at 15 min AI (ii) Upregulated 1–3 h AI with PL at 1–3 days AI	(i) Principal promoter of Wallerian degeneration (ii) Promotes immediate neutrophil recruitment to lesion site by the induction of adhesion molecules and IL-8 release (iii) iNOS induction in microglia, astrocytes, neutrophils, and monocytes	[118, 136, 137, 148, 182–184]

LIF	(i) Upregulated from 3 to 24 h AI	(i) Microglia/macrophage activator (ii) Overexpression increases macrophage/microglia proliferation and astrocytic activation	[24, 101]

IFN*γ*	(i) Detected from 1 to 12 h AI	(i) TCD8+ cytolytic response induction (ii) Principal inductor of MHCI (STAT1 phosphorylation) (iii) Promotes the following: (a) Proinflammatory cytokines and chemokines (b) CNS macrophage recruitment (c) Activation of CNS resident and infiltrating APC populations (iv) Low concentrations promote synaptic circuitry homeostasis	[118, 192]

IL-4	(i) High levels 24 h AI, concentrations remain during 7 days and decrease 3 days AI	(i) Neuroinflammatory regulation in various pathological conditions (ii) Confers regenerative properties to macrophages (iii) Controls free radical production in peripheral macrophages and microglia	[166, 193–199]

IL-13	(i) Detected 1 day AI	(i) Macrophage activation onto M2 phenotype	[166, 199]

IP-10/CXCL10	(i) Expressed locally 30 min AI, increases at 1 h, PL at 6 h. Remains increased up to 5 days and decreases to the baseline by day 14	(i) Recruits CD4 Th1 cells via CXCR3AR (ii) Inhibits angiogenesis, growth, and chemotaxis of endothelial cells via the CXCR3BR	[95, 184, 200]

IL-17	(i) Detected from 1 h AI, PL at 24 h, and barely detected up to 72 h AI	(i) Inflammation in autoimmune diseases	[201–204]

MIP-1*α*/CCL4	(i) Locally produced from 30 to 60 min AI, PL 3–6 h, decreases by 1 day and remains barely detected up to 24 days AI	(i) Macrophage infiltration	[200, 205–207]

MIP-1*β*	(i) Detected 3–6 days AI, remains barely detected up to (ii) 24 days AI	(i) Macrophage infiltration	[200, 205–207]

MCP1/CCL2	(i) Detected from 1 h AI with PL at 24 h and remains low up to 24 days AI	(i) Macrophage and PMN infiltration mediator	[106, 184, 200, 205, 206]

min: minutes; AI: after injury; PL: peak levels.

**Table 2:** Growth factors after SCI.

Item	Detection timeframe	Roles	Reference
NGF and NT-3	Detected 6 h AI	Neuron survival and regeneration AI	[208]

BDNF	Detected 6 h AI, increases up to 6 weeks, and decreases 12 weeks AI	(i) M2 macrophage phenotype induction (ii) Production of several extracellular matrix proteins for tissue remodeling and repair, neurotrophic support, and axonal regeneration (iii) Increases growth, angiogenic, and axonal guidance factors	[125, 150, 209, 210]

aFGF	Detected 1 h AI, PL 5–7 days, and remains elevated up to 14–21 days AI	(i) Potent mitogenic and chemotactic agent for vascular endothelial cells, dermal fibroblasts, and epidermal keratinocytes (ii) Promotes Th2 cytokine and neurotrophic factor production and M2 response	[209, 211]

bFGF	Detected 1 h AI, PL at day 3, remains elevated 5–7 days, and returns to low levels 14–21 days AI	(i) Angiogenesis	[209]

TGF-*β*	Detected at 24 h AI	(i) Immunosuppressant (ii) Tissue stabilization and structural preservation (iii) Neural regeneration and repair	[212]

min: minutes; AI: after injury; PL: peak levels.
